# Knowledge, Attitudes, and Practices of Community Pharmacists About Nutrition and Lifestyle Medications and Counseling in the Aseer Region of Saudi Arabia: A Cross-Sectional Study

**DOI:** 10.7759/cureus.85757

**Published:** 2025-06-11

**Authors:** Sultan M Alshahrani

**Affiliations:** 1 College of Pharmacy, King Khalid University, Abha, SAU

**Keywords:** community pharmacists, counseling, knowledge, lifestyle, nutrition, saudi arabia

## Abstract

Introduction: Community pharmacists possess a distinctive capability to provide lifestyle and nutrition counseling; however, their integration into public health systems in Saudi Arabia is still constrained.

Aim: The objective of this study was to evaluate the knowledge, attitudes, and practices (KAP) of community pharmacists in the Aseer region about lifestyle medications and nutritional counseling.

Methods: A cross-sectional study was performed, including 257 licensed community pharmacists utilizing a self-administered, structured questionnaire consisting of six items each on KAP. Descriptive statistics were employed to explore demographic characteristics and Likert-scale responses. Multivariable linear regression was conducted to determine predictors of attitude and practice scores.

Results: Participants exhibited moderate expertise, particularly excelling in BMI interpretation and drug-nutrient interactions, alongside generally positive attitudes; 174 respondents (67.7%) agreed that pharmacists can positively impact food habits. However, deficiencies in practice were noted where merely 54 (21.0%) indicated patient follow-up, and 105 (40.9%) regularly utilized BMI evaluation during counseling.

Conclusion: Although pharmacists demonstrate willingness and foundational knowledge to offer lifestyle counsel, actual implementation is constrained. Reforms in pharmacy education, ongoing training, and incorporation into national preventive healthcare initiatives are necessary. Empowering pharmacists is consistent with the objectives of Saudi Vision 2030 to enhance public health and chronic illness management.

## Introduction

Chronic diseases such as cardiovascular disease, obesity, and type 2 diabetes constitute key contributors to world mortality, much of which is controlled by lifestyle modifications [1]. Nutrition-related risk factors contribute to more fatalities globally than tobacco, highlighting the critical need to improve dietary practices and increase physical activity as health objectives [2]. Pharmacists, due to their accessibility and respected position within communities, are ideally situated to provide such interventions. However, the incorporation of nutrition and lifestyle counseling into standard pharmacy practice is still inconsistent and inadequately developed [3].

Recent research indicates that pharmacy professionals routinely offer dietary guidance, predominantly concerning diabetes (70%), cardiovascular problems (64%), and weight control (53%); nevertheless, they often lack formal training and confidence to deliver this advice appropriately [4]. A mixed-methods study revealed that, although the majority of pharmacists consider themselves accountable for offering such advice, a substantial deficiency exists in both knowledge and infrastructure to assist them. These findings correspond with increasing demands to integrate nutrition science more thoroughly into pharmacy courses and ongoing professional development frameworks [5,6].

Pharmacists in the UK and Europe have indicated a need for enhanced interprofessional collaboration with dietitians, access to current guidelines, and organized toolkits to facilitate lifestyle-related counseling [4,6]. Acar et al. [5] discovered that pharmacists regularly provide nutritional counseling for diabetes and weight management. Nevertheless, few felt adequately prepared to address sustainable diets, food-drug interactions, or micronutrient shortages with confidence [6]. The identified knowledge deficiencies reflect global apprehensions, including those in Saudi Arabia, where community pharmacy practitioners expressed a strong motivation to advise patients on nutrition but cited time limitations and insufficient formal training as significant obstacles [7].

A promising approach to addressing these deficiencies is an interprofessional, team-oriented lifestyle and nutrition course offered in the United States to pharmacy, dietetics, and medical students. The elective, based on the 5-2-1-0 behavioral framework (five servings of fruits and vegetables, two hours or less of screen time, one hour of physical activity, and zero sugary beverages), markedly enhanced students' confidence (20% increase) and knowledge (87% increase) in providing lifestyle coaching [8]. The results illustrate the practicality and advantages of integrating nutrition coaching training within first professional education.

Moreover, Alsabbah et al. revealed that, although 70% of Saudi pharmacy professionals expressed confidence in general dietary counseling, just 40% demonstrated confidence in therapeutic nutrition, and merely 35% in pediatric nutrition [7]. Obstacles include time constraints (65%) and inadequate knowledge (50%) were prevalent, indicating a pressing necessity for ongoing education and structural assistance. Comparable trends are observable in Australian, Jordanian, and Canadian studies, signifying that this is a worldwide concern [9-11].

Pharmacy education must include systematic instruction in public health nutrition and behavior change communication to leverage the pharmacist's accessibility and influence. This study aims to evaluate the knowledge, attitudes, and practices (KAP) of community pharmacists in the Aseer region of Saudi Arabia on lifestyle medications and nutrition counseling, to guide future curricular reforms and health policy initiatives.

## Materials and methods

Study design

This study utilized a cross-sectional quantitative methodology to evaluate the KAP of community pharmacists about lifestyle medications and counseling. A structured, self-administered questionnaire served as the principal instrument for data collection. The survey was disseminated among community pharmacists throughout the Aseer region of Saudi Arabia. The research was carried out from January to February 2025. The identity of participants and the confidentiality of responses were maintained throughout the study to encourage transparent self-reporting and mitigate response bias.

Study population

The target population consisted of licensed community pharmacists employed in both chain and independent pharmacies in urban and rural areas of the Aseer region. According to recent research [12], there are roughly 747 registered pharmacists in the Aseer region. Utilizing Raosoft’s online sample size calculator (www.raosoft.com/samplesize.html), a minimum sample size of 252 was determined, employing a 95% confidence level, a 5% margin of error, and a 50% response distribution. A total of 257 pharmacists completed the questionnaire, exceeding the necessary sample size and guaranteeing representative statistical power.

Study tool and data collection

The questionnaire employed in this study was designed based on previous research evaluating the KAP of pharmacy professionals about lifestyle medications and dietary counseling [4,7,10]. It was designed for self-administration and organized to promote clarity, ease of completion, and relevance to community pharmacy practice. A pilot study on 15 participants was conducted to evaluate the internal consistency and validity. Chronbach's alpha factor was calculated for 10 questions. It was equal to 0.782, which reflects an acceptable reliability level.

The knowledge area comprised six items assessing pharmacists' comprehension of lifestyle pharmaceuticals, encompassing their therapeutic function in chronic illness management, the influence of diet on health outcomes, and awareness of drug-nutrient interactions. These items were intended to assess the depth and precision of pharmacists' factual knowledge concerning lifestyle-oriented medication.

The attitude part included six statements that encapsulated pharmacists' attitudes and perceptions regarding their professional responsibility in advocating for healthy lifestyle choices. This section evaluated motivation, perceived accountability, and readiness to participate in lifestyle and nutrition counseling within the community pharmacy context.

The practice domain included six items designed to measure the frequency and consistency of pharmacists’ counseling behaviors. These items addressed how often pharmacists engaged in patient discussions related to diet, exercise, and other lifestyle modifications; their use of educational materials; and collaboration with other healthcare professionals in delivering preventive care.

Data analysis

All completed survey responses were reviewed for completeness and input into Statistical Product and Service Solutions (SPSS, version 26; IBM SPSS Statistics for Windows, Armonk, NY) for analysis. Descriptive statistics, including frequencies and percentages, were employed to represent demographic variables and item-level responses for the KAP domains. Responses to Likert-scale items were assigned values from 1 (Strongly disagree) to 5 (Strongly agree), and composite scores for each domain were derived by averaging the responses across the six relevant items.

Multiple linear regression analysis was performed to determine key variables of pharmacists' views and actions about nutrition and lifestyle counseling. Attitude and practice scores were analyzed as continuous dependent variables in two distinct models. The independent variables comprised gender, educational attainment (PharmD versus dachelor’s degree), years of experience (classified as 0-5, 6-10, and over 11 years), type of pharmacy (chain versus independent), practice setting (urban versus rural), and prior training in lifestyle or preventive medicine. The knowledge score was incorporated as an independent variable in both models, while the attitude score was utilized as a predictor in the model where practice served as the dependent variable. Regression coefficients, corresponding p-values, and 95% confidence intervals were presented for each predictor. A p-value below 0.05 was considered statistically significant.

Ethical considerations

Ethical approval was granted by the Research Ethics Committee of King Khalid University (KKU-24-2025-14). Informed consent was obtained electronically before survey access. All responses were anonymous, securely stored, and used solely for academic research purposes. Only the principal investigators had access to the dataset.

## Results

Participant characteristics

A total of 257 community pharmacists from the Aseer region participated in the study. The majority were male pharmacists (n = 164, 63.8%), aged 20-29 years (n = 144, 56.0%), and held a PharmD degree (n = 172, 66.9%). The majority of respondents were employed in chain pharmacies (n = 229, 89.1%) and resided in urban areas (n = 203, 79.0%). Concerning years of experience, 59.9% (n = 154) had 0-5 years, 24.9% (n = 64) had 6-10 years, and 15.2% (n = 39) had over 11 years. Furthermore, over 50% of the participants (n = 146, 56.8%) indicated that they have undergone training in nutrition and counseling (Table 1).

**Table 1:** Participants’ characteristics (n = 257)

Characteristic	Category	Frequency (n)	Percentage (%)
Gender	Male	164	63.80%
	Female	93	36.20%
Age Group	20–29 years	144	56.00%
	30–39 years	76	29.60%
	40+ years	37	14.40%
Education Level	Bachelor's degree	58	22.60%
	PharmD	172	66.90%
	Postgraduate degree	27	10.50%
Pharmacy Type	Chain pharmacy	229	89.10%
	Independent pharmacy	28	10.90%
Location	Urban	203	79.00%
	Rural	54	21.00%
Years of Experience	0–5 years	154	59.90%
	6–10 years	64	24.90%
	11+ years	39	15.20%
Have you ever received training on nutrition and its counseling	Yes	146	56.20%
	No	111	43.20%

Participants’ knowledge of nutrition and lifestyle medications

Overall, the participants have demonstrated a moderate level of knowledge. The most consensus was noted for the statements “I can identify drug-nutrient interactions” (n = 162, 63.0%) and “I am confident calculating and interpreting BMI” (n = 159, 61.9%). Awareness of dietary risks during pregnancy (n = 156, 60.7%) and adherence to national dietary standards (n = 153, 59.5%) exhibited considerable concurrence. Nonetheless, diminished familiarity was observed regarding the 5-2-1-0 framework, as merely 139 participants (54.1%) indicated awareness of its components (Table 2).

**Table 2:** Participants’ knowledge regarding nutritional medications and counseling

Knwoledge Statement	Strongly Agree n (%)	Agree n (%)	Neutral n (%)	Disagree n (%)	Strongly Disagree n (%)
I am familiar with national dietary guidelines.	62 (24.1%)	91 (35.4%)	48 (18.7%)	44 (17.1%)	12 (4.7%)
I understand lifestyle medications for hypertension and diabetes.	57 (22.2%)	87 (33.9%)	50 (19.5%)	52 (20.2%)	11 (4.3%)
I know the components of the 5-2-1-0 framework.	56 (21.8%)	83 (32.3%)	59 (23.0%)	48 (18.7%)	11 (4.3%)
I can identify drug–nutrient interactions.	71 (27.6%)	91 (35.4%)	48 (18.7%)	31 (12.1%)	16 (6.2%)
I am confident calculating and interpreting BMI.	73 (28.4%)	86 (33.5%)	48 (18.7%)	40 (15.6%)	10 (3.9%)
I understand dietary risks during pregnancy.	67 (26.1%)	89 (34.6%)	55 (21.4%)	36 (14.0%)	10 (3.9%)

Attitudes toward nutrition and lifestyle medications and counseling 

The participants' attitude responses demonstrated a general positivity. A significant percentage of participants concurred that pharmacists may positively impact eating habits (n = 174, 67.7%), that lifestyle counseling needs to be included in their job duties (n = 143, 55.6%), and that such interventions enhance patient outcomes (n = 145, 56.4%). Furthermore, 174 pharmacists (67.7%) endorsed the incorporation of lifestyle medicine into pharmacy education. Only 147 participants (57.2%) expressed confidence in providing nutrition counsel, highlighting the necessity for specialized training and support (Table 3).

**Table 3:** Participants’ attitudes regarding nutritional medications and counseling

Attitudes Statement	Strongly Agree n (%)	Agree n (%)	Neutral n (%)	Disagree n (%)	Strongly Disagree n (%)
Pharmacists should provide lifestyle counseling.	58 (22.6%)	85 (33.1%)	51 (19.8%)	49 (19.1%)	14 (5.4%)
Lifestyle counseling improves patient outcomes.	69 (26.8%)	76 (29.6%)	52 (20.2%)	41 (16.0%)	19 (7.4%)
I support integrating lifestyle medicine in pharmacy education.	76 (29.6%)	98 (38.1%)	50 (19.5%)	21 (8.2%)	12 (4.7%)
Pharmacists can influence dietary habits positively.	80 (31.1%)	94 (36.6%)	42 (16.3%)	31 (12.1%)	10 (3.9%)
Patients trust pharmacists for lifestyle advice.	62 (24.1%)	97 (37.7%)	46 (17.9%)	37 (14.4%)	15 (5.8%)
I feel confident providing nutrition guidance.	64 (24.9%)	83 (32.3%)	53 (20.6%)	36 (14.0%)	21 (8.2%)

Participants’ perception regarding practice and behavior toward nutritional medications and counseling 

Practice scores exhibited variability among items. The predominant activity conducted was counseling diabetes patients with diet (n = 175, 68.1%), followed by collaboration with physicians on lifestyle matters (n = 152, 59.1%) and by the distribution of leaflets or educational materials (n = 135, 52.5%). Nonetheless, less than half of the participants indicated that they consistently calculated BMI during counseling (n = 105, 40.9%) or advised hypertensive patients to engage in physical activity (n = 97, 37.7%). Significantly, merely 54 pharmacists (21.0%) indicated that they do follow-ups with patients subsequent to lifestyle counseling. These findings underscore a substantial gap between pharmacists’ positive attitudes and their actual implementation of lifestyle counseling practices (Table 4).

**Table 4:** Participants’ responses on practice questions regarding nutritional medications and counseling

Practice Statment	Strongly Agree n (%)	Agree n (%)	Neutral n (%)	Disagree n (%)	Strongly Disagree n (%)
I routinely counsel diabetic patients on diet.	71 (27.6%)	104 (40.5%)	37 (14.4%)	26 (10.1%)	19 (7.4%)
I recommend physical activity to hypertensive patients.	26 (10.1%)	71 (27.6%)	63 (24.5%)	57 (22.2%)	40 (15.6%)
I provide brochures on healthy eating.	64 (24.9%)	71 (27.6%)	76 (29.6%)	39 (15.2%)	7 (2.7%)
I assess weight status or BMI during counseling.	42 (16.3%)	63 (24.5%)	89 (34.6%)	40 (15.6%)	23 (8.9%)
I collaborate with physicians on lifestyle issues.	75 (29.2%)	77 (30.0%)	42 (16.3%)	38 (14.8%)	25 (9.7%)
I follow up with patients on lifestyle changes.	28 (10.9%)	26 (10.1%)	75 (29.2%)	66 (25.7%)	62 (24.1%)

Predictors of attitude and practice about nutrition counseling 

Multivariable linear regression analysis was conducted to identify predictors of pharmacists’ attitudes and practices toward nutrition and lifestyle counseling. As shown in Table 5, several variables were significantly associated with attitude scores. Female pharmacists demonstrated higher attitude scores compared to males (β = 1.103, p = 0.032), and those holding a PharmD degree scored significantly higher than those with a bachelor's degree (β = 1.573, p = 0.015). Conversely, fewer years of experience were negatively associated with attitudes. Pharmacists with 0-5 years and 6-10 years of experience had lower scores than those with more than 11 years (β = -2.494, p = 0.008; β = -1.882, p = 0.041, respectively). Prior training in nutrition or preventive medicine showed a strong positive association (β = 2.482, p < 0.001), as did higher knowledge scores (β = 0.635, p < 0.001). Pharmacy type and location were not significant predictors.

**Table 5:** Predictors of attitude and practice regarding nutrition counseling a: Gender: Male (reference category); b: Education: Bachelor’s degree (reference category); c: Years of experience: >11 years (reference category for 6–10 years comparison); d: Years of experience: >11 years (reference category for 0–5 years comparison); e: Pharmacy type: Independent pharmacy (reference category); f: Location: Rural (reference category); g: Training: No training (reference category)

Independent Variable	Attitude Coef.	p-value (Attitude)	95% CI Lower (A)	95% CI Upper (A)	Practice Coef.	p-value (Practice)	95% CI Lower (P)	95% CI Upper (P)
Intercept	27.894	<0.001	22.741	33.048	23.531	<0.001	18.42	28.642
Gender (Female vs Male)^a^	1.103	0.032	0.098	2.108	0.307	0.414	-0.431	1.045
Education (PharmD vs Bachelor)^b^	1.573	0.015	0.326	2.82	0.924	0.071	-0.078	1.926
Experience (6–10 vs 11+)^c^	-1.882	0.041	-3.678	-0.086	-0.785	0.227	-2.072	0.502
Experience (0–5 vs 11+)^d^	-2.494	0.008	-4.312	-0.675	-1.354	0.039	-2.643	-0.065
Pharmacy Type (Chain vs Independent)^e^	0.682	0.091	-0.112	1.476	0.244	0.307	-0.222	0.71
Location (Urban vs Rural)^f^	0.393	0.348	-0.431	1.218	0.072	0.709	-0.337	0.481
Training (Yes vs No)^g^	0.456	0.672	0.402	1.013	0.105	0.981	-0.018	1.192
Knowledge Score	0.635	<0.001	0.421	0.849	0.742	<0.001	0.529	0.955
Attitude Score	---	---	---	---	0.268	0.021	0.041	0.495

In the practice model, similar predictors were observed. Knowledge (β = 0.742, p < 0.001) and attitude scores (β = 0.268, p = 0.021) were both positively associated with higher practice scores. Pharmacists with fewer years of experience (0-5 years) reported significantly lower practice scores than those with 11+ years (β = -1.354, p = 0.039). No significant associations were found for gender, education, location, training, or pharmacy type in relation to practice.

Overall, the findings in Table 5 highlight the importance of professional training, clinical knowledge, and experience in shaping pharmacists' attitudes and engagement with nutrition-related counseling.

## Discussion

This research is one of the first studies in Saudi Arabia to analyze the KAP of community pharmacists about nutrition and lifestyle advice through a validated, multidimensional evaluation methodology. Our data indicate predominantly favorable attitudes and moderate knowledge levels among pharmacists, but also expose significant deficiencies in practice, especially with patient follow-up, counseling methodologies, and professional confidence. These findings have significant implications for public health delivery, pharmacist education, and national healthcare priorities, particularly in the context of Saudi Arabia’s Vision 2030 and anticipated upgrades in insurance coverage and community-based medical care.

In alignment with international studies, the majority of pharmacists in this study endorsed lifestyle counseling as part of their professional duties. However, their capacity to convert this mindset into persistent practice seems constrained. Comparable barriers have been recorded in research from Egypt and Jordan, where community pharmacists indicated low counseling frequencies despite acknowledging the significance of their role in public health nutrition [9,13]. This disconnection indicates that structural obstacles, including time limitations, inadequate training, and insufficient institutional support, can hinder the incorporation of nutrition care into pharmacy operations [14].

Educational background proved to be a substantial predictor of both attitude and practice scores, with PharmD holders exhibiting greater engagement than individuals possessing only a bachelor’s degree. This corresponds with studies from Iran and Canada, which indicated that pharmacists bearing clinically oriented degrees had more confidence and competence in delivering dietary supplement counseling [11,15]. These findings emphasize the necessity to update the pharmacy curriculum in Saudi Arabia to incorporate structured information on medical nutrition therapy, drug-nutrient interactions, and lifestyle management strategies [16].

Moreover, our regression analysis revealed that previous training in lifestyle or preventive medicine is a significant predictor of both positive attitudes and competent counseling practices. This study aligns with the results of Sunguya et al., who revealed that focused nutrition training markedly enhances healthcare workers' knowledge and practical skills [17]. This research revealed that trained pharmacists achieved superior scores in all KAP areas, thereby underscoring the significance of continuing professional development (CPD) programs in this field. The prevailing holistic perspective is that training enhances technical proficiency while promoting interdisciplinary teamwork and patient-centered communication skills [16,18].

From a policy perspective, community pharmacists are progressively acknowledged as key contributors to national public health measures. In nations including Nigeria and Ethiopia, pharmacists have been deployed to deliver frontline health education, pharmaceutical therapy management, and preventive screenings [14,19]. Saudi Arabia's Vision 2030 corresponds with this worldwide transformation, seeking to evolve toward a more prevention-focused and insurance-based healthcare paradigm. Expanding the role of community pharmacists in nutrition and lifestyle counseling could reduce the burden on primary care physicians and enhance chronic disease outcomes at the population level as part of this shift [20].

This expansion of roles necessitates the support of structural enablers. Research indicates that pharmacists frequently lack the necessary resources and institutional structures to deliver consistent health promotion services [21]. Incorporating nutrition into national health policy, establishing explicit referral paths to dietitians, and utilizing digital tools for nutritional assessment could augment pharmacists' efficacy and influence. Collaborative models incorporating pharmacists in extensive public health programs can markedly enhance patient participation and health literacy [22].

This study involves multiple limitations. The utilization of a self-administered questionnaire may induce response bias, especially in the reporting of attitudes and practices, potentially influenced by social desirability. The cross-sectional design restricts the capacity to deduce causal links between the identified predictors and pharmacists' behaviors. Third, while the sample size was statistically sufficient, participants were primarily sourced from the Aseer region, thus limiting the generalizability of the findings to other regions in Saudi Arabia. The sampling method may have unintentionally compensated more engaged or accessible pharmacists. Future research should adopt a more extensive national sample approach and integrate qualitative methodologies to provide a more profound understanding of implementation problems.

## Conclusions

This study provides a comprehensive evaluation of community pharmacists' KAP about nutrition and lifestyle counseling in Saudi Arabia. Pharmacists demonstrated largely positive attitudes and reasonable proficiency; however, practical application was insufficient. Notably, factors including prior training, high knowledge scores, and substantial professional experience were associated with improved attitudes and practices. These findings highlight an urgent need for capacity improvement in this domain.

Enhancing the pharmacy curriculum with structured education in nutrition, lifestyle pharmacotherapy, and patient-centered counseling is essential. The concurrent advancement of national continuing professional education (CPE) programs and the incorporation of pharmacists into public health initiatives will enable them to serve as primary health educators. This is especially important as Saudi Arabia advances towards Vision 2030, which prioritizes a healthcare paradigm centered on prevention, wellness, and insurance-based service provision. Investing in pharmacists' education, resources, and their integration into multidisciplinary care teams is crucial for enhancing chronic illness outcomes, alleviating demand on primary care, and advancing population health. To do this, policymakers, educators, and professional organizations must cooperate to enhance pharmacy practice and guarantee that pharmacists fulfill their complete potential in supporting national public health objectives.
